# Association of antioxidant status and inflammatory markers with metabolic syndrome in Thais

**DOI:** 10.1186/s41043-018-0158-9

**Published:** 2019-01-03

**Authors:** Kanjana Suriyaprom, Sarunya Kaewprasert, Pumpath Putpadungwipon, Pisit Namjuntra, Suwit Klongthalay

**Affiliations:** 1 Faculty of Medical Technology, Rangsit University, Paholyothin Road, Pathumthani, 12000 Thailand; 2 Department of Tropical Nutrition and Food Science, Faculty of Tropical Medicine, Mahidol University, 420/6 Rajvithi Road, Rajthevee, Bangkok, 10400 Thailand

**Keywords:** Metabolic syndrome, Antioxidant status, Inflammatory markers, Hematological parameters, Thai

## Abstract

**Background:**

An oxidant/antioxidant disequilibrium has been suggested as having a role in the pathogenesis of some diseases. Metabolic syndrome (MS) is significantly associated with cardiovascular disease and type 2 diabetes. The pathogenesis of MS is complex and not well understood. The purposes of the present study were to compare enzymatic and non-enzyme antioxidants, anthropometric, hematological, and biochemical findings between subjects with MS and without MS and to evaluate the relationship between antioxidant status and hematological parameters with the components of MS.

**Methods:**

Metabolic syndrome was assessed by using the modified National Cholesterol Education Program, Adult Treatment Panel III criteria. Three hundred Thais, 124 with MS and 176 without MS, were included in the study. Each subject was tested for erythrocyte superoxide dismutase (SOD), glutathione peroxidase, (GPX), catalase (CAT), albumin and vitamin C levels, and hematological findings.

**Results:**

Subjects with MS had lower SOD and CAT levels than those without MS (*p* < 0.01). Subjects with MS had lower vitamin C and albumin levels than those without MS (*p* < 0.05). The hematological findings were not significantly different between those with and without MS except the white blood cell (WBC) count which was significantly higher in those with MS. SOD and CAT levels were significantly positively associated with HDL-C levels and negatively associated with components of MS. After adjusting for potential covariates, we found lower SOD and vitamin C levels and higher WBC counts were significantly associated with MS (*p* < 0.05).

**Conclusions:**

These findings suggest an alteration in antioxidant status and an increase in inflammatory markers are associated with MS and its components among Thais; subjects with MS may be more likely to have oxidative stress problems.

## Background

Metabolic syndrome (MS) is an important public health problem, and its occurrence is increasing worldwide [1, 2]. It is estimated to affect 23% of the Thai population, and the prevalence is increasing possibly due to a transition in the population from a rural to an urban environment [3]. MS is a collection of risk factors predictive of future cardiovascular disease and diabetes mellitus [2]. The pathogenesis of MS and its components is complex and not well understood, although insulin resistance is considered to be a common factor linked to the other components of MS, including elevated plasma glucose, obesity, dyslipidemia, and hypertension [4]. Under physiological conditions, a dynamic equilibrium exists between the production of reactive oxygen species (ROS) and endogenous antioxidant defense. ROS are neutralized by antioxidant defense mechanisms. Antioxidant systems include antioxidant enzymes, such as catalase (CAT), glutathione peroxidase (GPX), and superoxide dismutase (SOD); and non-enzymatic antioxidants, such as vitamin C, vitamin E, glutathione, and albumin [5]. Oxidative stress is caused by an imbalance between oxidants and antioxidants and has also been associated with some diseases [5, 6]. Inflammation is a manifestation of oxidative stress, and the pathways that generate the mediators of inflammation are all induced by oxidative stress [7]. Inflammation is also the most frequently cited mechanism for increased white blood cell (WBC) counts [8]. Albumin is a well-known acute phase protein that acts as a marker of inflammation and has antioxidant properties [9]. However, there is conflicting evidence regarding the association between antioxidant status in MS: some authors have found an association [10, 11] and others have not [12, 13]. Information regarding antioxidant status and the hematological-biochemical parameters found in MS is limited, especially from developing countries such as Thailand. This information about Thais with MS could assist efforts to reduce the risk of chronic disease, such as cardiovascular disease and diabetes. The purposes of the present study were to compare enzymatic and non-enzyme antioxidants, anthropometric, hematological, and biochemical findings between subjects with and without MS and to investigate possible associations among these enzymatic and non-enzymatic antioxidants and hematological parameters with the components of MS.

## Methods

### Subjects

The study protocol was approved by the Ethics Committee of Rangsit University (RSEC no.016/53), Thailand and in accordance with the Declaration of Helsinki. All subjects gave written informed consent prior to participation in the study. This case-control study enrolled 300 Thai volunteers (133 males, 167 females) aged 30–59 years from suburban and urban Bangkok, Thailand. Among them, 176 subjects without MS (77 males, 99 females) and 124 subjects with MS (56 males, 68 females) were chosen during the health screening program among check-up subjects in Medical Technology Clinic of Rangsit University between March 2012 and February 2013. The statistical power in our sample size calculation was 80% at alpha = 0.05, and the response rate of our study was 96.8%. We conducted a physical examination and obtained a medical history on all study subjects. Exclusion criteria for study subjects were a history of liver, kidney, inflammatory, gastrointestinal, respiratory, or cardiovascular diseases or consuming antioxidant vitamin supplements. Moreover, chi-square test (*p* > 0.05) found no significant difference in smoking status between the MS group (smoking 13.7%, non-smoking 86.3%) and non-MS group (smoking 10.2%, non-smoking 89.8%). MS was defined using modified National Cholesterol Education Program/Adult Treatment Panel III (NCEP/ATP III) criteria [14]. Subjects were diagnosed with MS if they had 3 of the following 5 factors: (1) impaired fasting glucose (≥ 100 mg/dl); (2) elevated blood pressure (systolic blood pressure ≥ 130 mmHg or diastolic blood pressure ≥ 85 mmHg); (3) having to a low high-density lipoprotein cholesterol (HDL-C) < 40 mg/dl in men or < 50 mg/dl in women; (4) hypertriglyceridemia (≥ 150 mg/dl); (5) abdominal obesity (waist circumference (WC) ≥ 90 cm in men and ≥ 80 cm in women). Some ethnic groups as Asians, particularly South Asians, appear to have proved to development of the MS at waist circumference lower than the NCEP/ATP III cut-off points. Therefore, specific value of Asians for waist circumference cut-off point was applied in this study.

### Anthropometric measurements

Height, weight, and waist circumference were measured for all subjects. Height measurement was typically taken two times and measured to the nearest 0.1 cm using a standard medical measuring rod. Weight was measured to the nearest 0.1 kg while the subject was standing upright position in bare feet and wearing lightweight clothing, using a body composition analyzer with software (Tanita body composition analyzer SC-330; Tanita, Tokyo, Japan). A body mass index (BMI) was calculated as weight in kilograms divided by height in meters squared. Waist circumference (WC) was measured with a flexible standard tape certified by the Ministry of Commerce, Thailand. Technique for measuring WC was essential to obtaining reliable data and the exact location for measuring WC in this study was the middle of the lower ribs and upper iliac crest. WC measurement was typically taken three times and measured to the nearest 0.1 cm by training technicians while the subject was standing upright position in bare feet after the subject exhaled. Blood pressure (BP) was measured at the right arm using an automatic blood pressure monitor (BP A2 Basic; Microlife AG, Widnau, Switzerland) after the subject rested in a sitting position for 5–10 min.

### Laboratory measurements

10 ml of venous blood were obtained from each subject in the morning after an overnight fast and checked for glucose, total cholesterol (TC), triglycerides (TG), high-density lipoprotein cholesterol (HDL-C), and albumin levels using a DADE Dimension AR®. Low-density lipoprotein cholesterol (LDL-C) was estimated using the Friedewald formula (LDL-C = TC − (HDL-C) − (TG/5)). White blood cell (WBC), red blood cell (RBC), and platelet counts, and hemoglobin (Hb) and hematocrit (Hct) levels were determined using a COULTER® Hematology Analyzer. Erythrocyte indices (mean corpuscular volume (MCV), mean corpuscular hemoglobin (MCH), and mean corpuscular hemoglobin concentration (MCHC)) were calculated according to Dacie and Lewis [15]. Serum vitamin C levels were determined following the method described by Liu et al. [16].

SOD levels were measured using a Randox test combination (Randox Laboratories, Ltd., United Kingdom). Xanthine and xanthine oxidase were used to generate superoxide radicals that react with 2-(4-iodophenyl) 3-(4-nitrophenol)-5 phenyltetrazolium chloride (INT) to form a red formazan dye. SOD activity was then measured by the degree of inhibition of this reaction. Results were expressed as U SOD/g Hb. The GPX level was determined using a Randox test combination (Randox Laboratories, Ltd., UK). GPX function is to catalyze the oxidation of reduced glutathione (GSH) to oxidized glutathione (GSSG) using t-butyl hydroperoxide. In the presence of glutathione reductase and NADPH, oxidized glutathione is immediately converted to the reduced form with a concomitant oxidation of NADPH to NADP^+^. The results are expressed as U GPX/g Hb. The CAT level was assessed according to Aebi [17]. CAT catalyzes the breakdown of hydrogen peroxide into water and oxygen; the rate of decomposition of hydrogen peroxide was monitored spectrophotometrically at a wavelength of 230 nm; CAT activity is expressed as U CAT/g Hb.

### Statistical analysis

Statistical analysis was performed using SPSS for Windows, version 11.5 (SPSS, Chicago, IL, USA). Medians with 95% confidence intervals (CI) were calculated. The differences between two groups were compared with the Mann-Whitney *U* test. Spearman rank was used to calculate correlations among variables. We used logistic regression analysis to assess links between MS as a dependent variable and other potential factors. The results were considered statistically significant at a *p* value < 0.05.

## Results

This study enrolled 300 Thai volunteers aged between 30 and 59 years. The prevalences of MS in our study subjects seen using modified NCEP ATP III definitions among study subjects are shown in Table 1. The most common component of MS seen in our study was low HDL-C (found in 64.7%). Anthropometric and biochemical results of subjects with and without MS are shown in Table 2. Chi-squared test (*p* > 0.05) found no large difference in sex composition between the MS group (male 45.2%, female 54.8%) and non-MS group (male 43.8%, female 56.2%). Subjects with MS had significantly higher mean values for weight, WC, BMI, blood pressure, glucose, TC, and TG and lower HDL-C values than those without MS (*p* < 0.01). No significant differences in hematological parameters were observed between these two groups, except for WBC count. The WBC count was significantly higher in those with MS than those without MS. Antioxidant findings for the two groups are shown in Table 3. Subjects with MS had significantly lower SOD and CAT levels than those without MS (*p* < 0.01). Vitamin C and albumin levels were also significantly lower in those with MS than those without MS (*p* < 0.05). The correlation coefficient for the components of MS and the studied variables are shown in Table 4. The SOD and CAT levels were significantly positively associated with HDL-C level and negatively associated with glucose, TG, BP, and WC levels. The WBC and RBC counts were significantly positively associated with TG, BP, and WC. Albumin was significantly negatively associated with BP and WC measurements. BMI was significantly positively associated with the WBC count and negatively associated with SOD, GPX, vitamin C, and albumin levels (*p* < 0.01). Table 5 shows the unadjusted and adjusted odds ratio (OR) for association with MS. Individuals with low SOD (≤ 884.2 U/g Hb) [18] and vitamin C levels (≤ 5 mg/l) [19] were 2.39 and 2.33 times more likely to have MS than those with normal SOD and vitamin C levels, respectively, after controlling for age, sex, and BMI. Individuals with high WBC levels (≥ 9.4 × 10^9^/l) [20] were 2.15 times more likely to have MS than those with a low WBC level after adjusting for potential covariates. However, we did not find an association between MS and CAT (≤ 19.2 × 10^4^ U/g Hb) [21] or GPX (≤ 15.96 U/g Hb) levels [21] using logistic regression analysis (*p* > 0.05).

**Table 1:** Prevalences of components of MS among all study subjects

Characteristics	Subjects without MS (*n* = 176)	Subjects with MS (*n* = 124)	Total (*n* = 300)
	*n* (%)	*n* (%)	n (%)
High WC	68 (38.6)	97 (78.2)	165 (55.0)
High TG level	19 (10.8)	66 (53.2)	85 (28.3)
Low HDL-C level	91 (51.7)	103 (83.1)	194 (64.7)
High BP	13 (7.4)	71 (57.3)	84 (28.0)
High fasting glucose level	3 (1.7)	41 (33.1)	44 (14.7)

High waist circumference (WC) ≥ 90 cm in men and ≥ 80 cm in women; high triglycerides (TG) level (≥ 150 mg/dl); low high-density lipoprotein cholesterol (HDL-C) < 40 mg/dl in men or < 50 mg/dl in women; high blood pressure (systolic blood pressure ≥ 130 mmHg or diastolic blood pressure ≥ 85 mmHg); high fasting glucose (≥ 100 mg/dl)

**Table 2:** Various studied factors among study subject with and without MS

Studied factor	Subjects without MS (*n* = 176)	Subjects with MS (*n* = 124)	*p* value
	Median (95% CI)	Median (95% CI)	
Age (years)	40.0 (38.0–42.0)	42.0 (39.0–43.0)	0.367
Weight (kg)	62.5 (60.7–65.7)	78.5 (76.0–80.1)	< 0.001*
BMI (kg/m^2^)	25.4 (23.8–26.2)	29.7 (28.1–31.1)	< 0.001*
WC (cm)	79.0 (77.8–81.0)	93.0 (90.0–96.0)	< 0.001*
Systolic BP (mmHg)	119.0 (114.0–120.0)	136.0 (133.0–140.0)	< 0.001*
Diastolic BP (mmHg)	78.0 (77.0–80.0)	90.0 (90.0–91.0)	< 0.001*
Glucose (mg/dl)	82.0 (80.0–83.0)	91.0 (89.0–95.0)	< 0.001*
TC (mg/dl)	204.0 (200.0–211.0)	223.0 (214.0–231.0)	< 0.001*
HDL-C (mg/dl)	52.0 (49.0–55.0)	48.0 (45.0–51.0)	< 0.001*
LDL-C (mg/dl)	130.0 (125.0–139.0)	138.0 (131.0–148.0)	0.065
TG (mg/dl)	83.0 (75.0–93.0)	158.0 (140.0–168.0)	< 0.001*
WBC count (× 10^9^/l)	6.5 (6.2–6.8)	7.5 (7.1–7.9)	< 0.001*
Platelet count (× 10^9^/l)	297.0 (284.0–307.5)	285.5 (269.9–301.2)	0.687
RBC count (× 10^12^/l)	4.8 (4.7–4.9)	4.9 (4.8–5.1)	0.262
Hemoglobin (g/dl)	13.4 (13.2–13.6)	13.5 (13.3–13.8)	0.189
Hematocrit (%)	40.9 (39.6–41.1)	41.0 (40.0–42.0)	0.177
MCV (fl)	83.5 (82.4–84.5)	82.9 (81.8–83.9)	0.115
MCH (pg)	28.4 (27.8–28.8)	28.1 (27.2–28.6)	0.283
MCHC (g/dl)	33.8 (33.6–34.0)	33.8 (33.6–33.9)	0.980

*BMI* body mass index, *BP* blood pressure, *TG* triglycerides, *TC* total cholesterol, *HDL-C* high-density lipoprotein cholesterol, *LDL-C* low-density lipoprotein cholesterol, *WBC* white blood cell, *RBC* red blood cell, *MCV* mean corpuscular volume, *MCH* mean corpuscular hemoglobin, *MCHC* mean corpuscular hemoglobin concentration

*p < 0.001, by Mann-Whitney *U* test

**Table 3:** Antioxidant levels among the subjects with and without MS

	Subjects without MS *n* = 176	Subjects with MS *n* = 124	*p* value
	Median (95% CI)	Median (95% CI)	
SOD (U/g Hb)	2129 (1892–2540)	1341 (1168–1711)	< 0.001*
GPX (U/g Hb)	31.1 (29.3–35.9)	28.1 (23.7–30.8)	0.140
CAT × 10^4^ (U/g Hb)	18.1 (17.6–19.3)	16.0 (15.2–18.0)	0.009*
Vitamin C (mg/dl)	6.7 (6.0–7.5)	5.9 (4.8–6.7)	0.034**
Albumin (g/dl)	4.5 (4.4–4.5)	4.4 (4.3–4.4)	0.002*

*CAT* catalase, *GPX* glutathione peroxidase, *SOD* superoxide dismutase

**p < 0.05, *p < 0.01 by Mann-Whitney *U* test

**Table 4:** Correlation coefficients for components of MS and the studied variables among all study subjects

	SOD	CAT	GPX	Vitamin C	Albumin	WBC count	RBC count
Glucose	− 0.198*	− 0.133**	0.071	− 0.033	0.052	0.078	0.103
TG	− 0.217*	− 0.241*	− 0.064	− 0.101	− 0.099	0.283*	0.150**
HDL-C	0.137**	0.190*	0.031	0.071	− 0.073	− 0.071	− 0.140**
Systolic BP	− 0.165*	− 0.159**	0.015	− 0.053	− 0.185*	0.207*	0.182*
Diastolic BP	− 0.156**	− 0.085	− 0.043	− 0.102	− 0.151*	0.200*	0.135**
WC	− 0.229*	− 0.201*	− 0.106	− 0.212*	− 0.151*	0.355*	0.229*
BMI	− 0.289*	− 0.123	− 0.298*	− 0.230*	− 0.223*	0.389*	0.078

*CAT* catalase, *GPX* glutathione peroxidase, *SOD* superoxide dismutase, *WBC* white blood cell, *RBC* red blood cell, *BP* blood pressure, *TG* triglycerides, *HDL-C* high-density lipoprotein cholesterol, *WC* waist circumference

**p < 0.05, *p < 0.01 by Spearman’s rank correlation (two-tailed)

**Table 5:** Odds ratios for association with MS using logistic regression analysis (*n* = 300)

Variable	Unadjusted OR (95% CI)	Adjusted OR^a^ (95% CI)
Vitamin C ≤ 5 (mg/l)^(19)^	2.38* (1.39–3.98)	2.33* (1.31–4.04)
SOD ≤ 884.2 (U/g Hb)^(18)^	3.09* (1.72–5.56)	2.39* (1.27–4.51)
GPX ≤ 15.96 (U/g Hb)^(21)^	1.83 (0.88–3.77)	0.56 (0.35–1.25)
CAT ≤ 19.2 × 10^4^ (U/g Hb)^(21)^	1.17 (0.73–1.88)	1.01 (0.59–1.71)
WBC ≥ 9.4 (10^9^/l)^(20)^	2.98* (1.41–4.89)	2.15** (1.01–4.10)

*CAT* catalase, *GPX* glutathione peroxidase, *SOD* superoxide dismutase, *WBC* white blood cell, *BMI* body mass index, *OR* odds ratio, *95% CI* 95% confidence interval

^a^Adjusted to the covariates age, gender, and BMI

**p < 0.05, *p < 0.01 using logistic regression analysis

Each cut-off points had cited from the reference number 18-21

## Discussion

Thailand has gone through a transition in the population from a rural to an urban environment, and the prevalence of MS is increasing in Thai population [3]. However, the mechanisms causing the development and progression of MS are still unclear. We found significant differences in some studied enzyme and non-enzyme antioxidants and inflammation markers between subjects with and without MS. The most common component of MS among our study subjects with MS was a low HDL-C (64.7%), even though the most important component of MS in the literature is insulin resistance [4, 22]. Our findings are similar to those of Aekplakorn et al. [3] and Nillakupt et al. [23]. HDL particles have also an important role in anti-oxidative stress by reducing oxidative modifications of LDL and preventing accumulation of lipoperoxides in LDL [24]. Central obesity (55.0%) defined using WC was also common components of MS found in our subjects and obesity is a chronic inflammatory state and related to increased free radical concentrations [25]. Furthermore, 14.7% of subjects in this study had impaired glucose metabolism, and Choi et al. [26] reported that high glucose can mediate production of ROS and oxidative stress. Our findings suggest obesity, dyslipidemia, and abnormalities of glucose metabolism may promote oxidative stress.

Oxidative stress is caused by the imbalance between oxidants and antioxidants. Increased oxidative stress contributes to impaired vascular function, inflammation, and atherosclerosis [6, 10]. In this study, we investigated enzymatic and non-enzymatic antioxidant defense systems that are directly involved in the neutralization of ROS. We hypothesized that an alteration in antioxidant status may be reduced in MS, and our findings support this hypothesis. Our key findings in Thai population support the fact that individuals with MS may be more susceptible to oxidative stress resulted in alterations of antioxidant defense systems. This is in agreement with Chen et al. [10] and Yokota et al. [11]. We found significantly lower SOD and CAT levels among Thai subjects with MS, similar to a study from Taiwan [10] and Japanese subjects [11]. SOD is an endogenous free-radical scavenger against superoxide, and these enzymes dismute superoxide radicals to hydrogen peroxide and oxygen. CAT is the enzyme associated with hydrogen peroxide destruction, partly generated by SOD [5]. According to the mechanism of CAT during lengthy exposure to ROS, Kirkman et al. [27] reported in their study that the CAT-bound NADPH became oxidized to NADP^+^ and CAT activity fell to one third of the initial activity. Moreover, our findings from logistic regression analysis also support that the decreased activity of SOD and vitamin C increased risk of MS more than two-fold. Therefore, our results indicate that decreased antioxidants may be suggestive of increased utilization of free radical scavenging system to combat the ROS. However, no changes in SOD or CAT levels were seen in subjects with MS compared with controls in a study from Spain [12] or from Poland [13]. The differences between our results and those of previous studies regarding the associations between antioxidant enzymes and MS may result from different lifestyle and genetic backgrounds of study populations.

Additionally, our study also confirms that antioxidant activities are associated with components of MS. Our findings are in line with Mansengo et al. [28] that found a negative association between SOD and CAT levels and hypertension. Antioxidants should have beneficial effects on hypertension control, and reduction of oxidative damage should result in a reduction in blood pressure [29]. Abdominal obesity is a component of MS. WC was significantly negatively associated with SOD and CAT levels in our study, similar to the findings of Amirkhizi et al. [30]. Obesity is associated with an oxidative burden that can be related to a reduction in antioxidant enzyme activity [25]. Therefore, the results of our study suggest that metabolic risk factors have related to promoting oxidative stress in subjects with MS.

Albumin and vitamin C are part of antioxidant properties in plasma. In this study, vitamin C and albumin levels were significantly lower in subjects with MS than in subjects without MS, similar to the findings in Poland [31] and the USA [32]. Albumin exhibits free radical-trapping activity, and more than 70% of this activity in the serum was due to albumin. Albumin contains one reduced cysteine residue (Cys34), and with this is able to scavenge hydroxyl radicals [33]. Moreover, our results are also consistent with García et al. [34] who found vitamin C levels were inversely associated with markers of obesity. Central obesity is a common component of MS and has related to increased oxidative stress [25]. Data from USA population found that lower consumption of fruits and vegetables may have contributed to the reduced concentrations of vitamin C among participants with MS [32]. Thus, our results suggest that the lower antioxidant concentrations among MS subjects may have resulted from increased use of antioxidants, lower intakes of antioxidants, or both. MS subjects should become aware of low antioxidant levels in body by increasing consumption of an antioxidant diet. Further study should determine the antioxidant intakes with serum antioxidant concentrations in MS.

Inflammation is the frequently cited mechanism for an increased WBC count. WBC is activated resulting in the release of ROS and other mediators such as inflammatory cytokines [35]. Vitamin C has been found to suppress the inflammatory response by inhibiting NF-κB activity [36]. NF-κB is an important transcription factor that mediates inflammatory cytokines. In our study, subjects with MS had higher WBC counts and lower vitamin C levels than subjects without MS. Our results are similar to Wang et al. [37] who found elevated WBC counts were associated with components of MS in Chinese population from Taiwan. Chen et al. [10] also found subjects with MS had increased inflammation as seen in our study. One possible explanation for association of an increased WBC count with MS is that increased WBC may reflect the cytokine system. Because cytokines, especially IL-6, are the potent WBC differentiation and production factors produced mostly in adipose tissues [38], activated white blood cells produce multiple inflammatory cytokines that can impair insulin sensitivity on adipocytes and muscle cells [39], and this may also be linked to the pathophysiology of MS. Therefore, the results of our study suggest that subjects with MS may be more likely to have inflammation.

There are a few limitations of our study. First, the sample size was relatively small, so a prospective study should be undertaken to confirm the existence of a relationship between the antioxidant and inflammatory status and MS in larger sample size. Second, assessment of dietary antioxidant intake was not measured in the target population in this study. Therefore, a combined analysis of the relationships among dietary antioxidants, serum antioxidants, and MS may provide a more comprehensive understanding of this topic.

## Conclusions

In conclusion, in our study, some antioxidant levels and inflammatory markers were associated with MS and its components. These findings suggest subjects with MS may be more likely to have oxidative stress leading to lower antioxidant levels to compensate for higher ROS levels.

## Abbreviations

BP: Blood pressure
CAT: Catalase
CI: Confidence intervals
GPX: Glutathione peroxidase
GSSG: Oxidized glutathione
Hb: Hemoglobin
Hct: Hematocrit
HDL-C: High-density lipoprotein cholesterol
LDL-C: Low-density lipoprotein cholesterol
MCH: Mean corpuscular hemoglobin
MCHC: Mean corpuscular hemoglobin concentration
MCV: Mean corpuscular volume
MS: Metabolic syndrome
NCEP/ATP III: National Cholesterol Education Program/Adult Treatment Panel III
RBC: Red blood cell
ROS: Reactive oxygen species
SOD: Superoxide dismutase
TG: Triglycerides
WBC: White blood cell
WC: Waist circumference

## References

[1] Mozumdar A, Liguori G (2011). Persistent increase of prevalence of metabolic syndrome among U.S. adults: NHANES III to NHANES 1999–2006. Diabetes Care.

[2] Kaur J (2014). A comprehensive review on metabolic syndrome. Cardiol Res Pract.

[3] Aekplakorn W, Kessomboon P, Sangthong R (2011). Urban and rural variation in clustering of metabolic syndrome components in the Thai population: results from the fourth National Health Examination Survey 2009. BMC Public Health.

[4] Ruderman NB, Carling D, Prentki M, Cacicedo JM (2013). AMPK, insulin resistance, and the metabolic syndrome. J Clin Invest.

[5] Roberts CK, Sindhu KK (2009). Oxidative stress and metabolic syndrome. Life Sci.

[6] De Marchi E, Baldassari F, Bononi A, Wieckowski MR, Pinton P (2013). Oxidative stress in cardiovascular diseases and obesity: role of p66Shc and protein kinase C. Oxidative Med Cell Longev.

[7] Bryan S, Baregzay B, Spicer D, Singal PK, Khaper N (2013). Redox-inflammatory synergy in the metabolic syndrome. Can J Physiol Pharmacol.

[8] Farhangi MA, Keshavarz SA, Eshraghian M, Ostadrahimi A, Saboor-Yaraghi AA (2013). White blood cell count in women: relation to inflammatory biomarkers, hamatological profiles, visceral adiposity, and other cardiovascular risk factors. J Health Popul Nutr.

[9] Roche M, Rondeau P, Singh NR, Tarnus E, Bourdon E (2008). The antioxidant properties of serum albumin. FEBS Lett.

[10] Chen SJ, Yen CH, Huang YC, Lee BJ, Hsia S, Lin PT (2012). Relationships between inflammation, adiponectin, and oxidative stress in metabolic syndrome. PLoS One.

[11] Yokota T, Kinugawa S, Yamato M (2013). Systemic oxidative stress is associated with lower aerobic capacity and impaired skeletal muscle energy metabolism in patients with metabolic syndrome. Diabetes Care.

[12] Cardona F, Tu’nez I, Tasse I, Montilla P, Collantes E, Tinahones FJ (2008). Fat overload aggravates oxidative stress in patients with the metabolic syndrome. Eur J Clin Investig.

[13] Ziobro A, Duchnowicz P, Mulik A, Koter-Michalak M, Broncel M (2013). Oxidative damages in erythrocytes of patients with metabolic syndrome. Mol Cell Biochem.

[14] Alberti KG, Eckel RH, Grundy SM (2009). Harmonizing the metabolic syndrome: a joint interim statement of the International Diabetes Federation Task Force on Epidemiology and Prevention National Heart, Lung, and Blood Institute American Heart Association World Heart Federation International Atherosclerosis Society and International Association for the Study of obesity. Circulation.

[15] Dacie JV, Lewis SM (2007). Practical haematology.

[16] Liu TZ, Chin N, Kiser MD, Bigler WN (1982). Specific spectrophotometry of ascorbic acid in serum or plasma by use of ascorbate oxidase. Clin Chem.

[17] Aebi H (1984). Catalase in vitro. Methods Enzymol.

[18] Bogdanska JJ, Korneti P, Todorova B (2003). Erythrocyte superoxide dismutase, glutathione peroxidase and catalase activities in healthy male subjects in Republic of Macedonia. Bratisl Lek Listy.

[19] Harnroongroj T, Jintaridhi P, Vudhivai N (2002). B vitamins, vitamin C and hematological measurements in overweight and obese Thais in Bangkok. J Med Assoc Thail.

[20] Al-Gaithy ZK (2012). Clinical value of total white blood cells and neutrophil counts in patients with suspected appendicitis: retrospective study. World J Emerg Surg.

[21] Viroonudomphol D, Pongpaew P, Tungtrongchitr R (2000). Erythrocyte antioxidant enzymes and blood pressure in relation to overweight and obese Thai in Bangkok. Southeast Asian J Trop Med Public Health.

[22] Lebovitz HE (2001). Insulin resistance: definition and consequences. Exp Clin Endocrinol Diabetes.

[23] Nillakupt K (2010). Viravathana N. a survey of metabolic syndrome and its components in Thai medical cadets. J Med Assoc Thail.

[24] Mackness MI, Arrol S, Abbott C, Durrington PN (1993). Protection of low-density lipoprotein against oxidative modification by high-density lipoprotein associated paraoxonase. Atherosclerosis.

[25] Karaouzene N, Merzouk H, Aribi M, Merzouk SA, Berrouiguet AY, Narce M (2011). Effects of the association of aging and obesity on lipids, lipoproteins and oxidative stress biomarkers: a comparison of older with young men. Nutr Metab Cardiovasc Dis.

[26] Choi SW, Benzie IF, Ma SW, Strain JJ, Hannigan BM (2008). Acute hyperglycemia and oxidative stress: direct cause and effect?. Free Radic Biol Med.

[27] Kirkman HN, Galiano S, Gaetani GF (1987). The function of catalase-bound NADPH. J Biol Chem.

[28] Mansengo ML, Redon J, Martinez-Hervas S (2011). Different impacts of cardiovascular risk factor on oxidative stress. Int J Mol Sci.

[29] Baradaran A, Nasri H, Rafieian-Kopaei M (2014). Oxidative stress and hypertension: possibility of hypertension therapy with antioxidants. J Res Med Sci.

[30] Amirkhizi F, Siassi F, Djalali M, Shahraki SH (2014). Impaired enzymatic antioxidant defense in erythrocytes of women with general and abdominal obesity. Obes Res Clin Pract.

[31] Godala M, Materek-Kuśmierkiewicz I, Moczulski D (2015). Physical activity in patients with symptoms of metabolic syndrome reduces the concentration of plasma antioxidant vitamins-protective effect of vitamin C. Pol Merkur Lekarski.

[32] Ford ES, Mokdad AH, Giles WH, Brown DW (2003). The metabolic syndrome and antioxidant concentrations: findings from the Third National Health and Nutrition Examination Survey. Diabetes.

[33] Gutteridge JM (1986). Antioxidant properties of the proteins caeruloplasmin, albumin and transferrin. A study of their activity in serum and synovial fluid from patients with rheumatoid arthritis. Biochim Biophys Acta.

[34] García OP, Ronquillo D, del Carmen Caamaño M, Martínez G, Camacho M, Rosado JL (2013). Zinc, iron and vitamins A, C and e are associated with obesity, inflammation, lipid profile and insulin resistance in Mexican school-aged children. Nutrients.

[35] Panek J, Kuśnierz-Cabala B, Dolecki M, Pietron J (2012). Serum proinflammatory cytokine levels and white blood cell differential count in patients with different degrees of severity of acute alcoholic pancreatitis. Pol Przegl Chir.

[36] Bowie AG, O’Neill LAJ (2000). Vitamin C inhibits NF-kB activation by TNF via the activation of p38 mitogen-activated protein kinase. The J Immunol.

[37] Wang YY, Lin SY, Liu PH, Cheung BM, Lai WA (2004). Association between hematological parameters and metabolic syndrome components in a Chinese population. J Diabetes Complicat.

[38] Jonas MI, Kurylowicz A, Bartoszewicz Z (2015). Interleukins 6 and 15 levels are higher in subcutaneous adipose tissue, but obesity is associated with their increased content in visceral fat depots. Int J Mol Sci.

[39] Fernandez-Real JM, Vayreda M, Richart C (2001). Circulating interleukin 6 levels, blood pressure, and insulin sensitivity in apparently healthy men and women. J Clin Endocrinol Metab.

